# Pragmatic online obesity treatment in primary care: a hybrid randomized clinical trial of implementation strategies

**DOI:** 10.1038/s44325-024-00030-w

**Published:** 2024-11-06

**Authors:** Carly M. Goldstein, Emily Panza, Jacqueline F. Hayes, J. Graham Thomas, Kevin O’Leary, Rena R. Wing

**Affiliations:** 1 Weight Control and Diabetes Research Center, The Miriam Hospital, Providence, RI USA; 2 Department of Psychiatry and Human Behavior, Warren Alpert Medical School of Brown University, Providence, RI USA

**Keywords:** Health care, Endocrine system and metabolic diseases

## Abstract

Online behavioral weight loss (BWL) in primary care is effective and disseminable. This trial compared two implementation approaches on program uptake, use, and weight loss via a pragmatic hybrid type 2 implementation-effectiveness design to evaluate online BWL implementation (Rx Weight Loss [RxWL]) and effectiveness. This manuscript presents the implementation results. RxWL was implemented across a state-wide network of primary care clinics using lower- and higher-intensity implementation strategies (*Basic* [base program] and *Enhanced* [base plus enhanced training and dashboard], respectively) between 2018 and 2022. Nurse care managers (NCMs; *N* = 23) were recruited and block-randomized to implementation condition. Adult primary care patients (body mass index [BMI] > 25 kg/m^2^, internet-connected device access) were referred and enrolled by their NCMs. Outcomes were the proportion of eligible patients who enrolled in and completed RxWL by NCM condition, initial weight loss and regain over 12 and 24 months by NCM condition, and clinician acceptability and feasibility. NCMs (*N* = 12 *Enhanced*, *N* = 11 *Basic*) in *Enhanced* enrolled more patients (*N* = 490) than in *Basic* (*N* = 164). Although the proportion of patients who completed RxWL and mean weight loss did not differ by condition, different enrollment rates resulted in the *Enhanced* condition engaging more patients. NCMs rated RxWL as acceptable and feasible with no difference by condition. Findings support connecting primary care patients with technology-based health behavior change programs. Clinical trial registration number: ClinicalTrials.Gov identifier NCT03488212: https://clinicaltrials.gov/ct2/show/NCT03488212. Trial registration: clinicaltrials.gov Identifier: https://clinicaltrials.gov/study/NCT03488212.

## Introduction

High rates of obesity in the U.S. are driven by multi-level determinants that operate at the societal level (e.g., food industry/policy), the community level (e.g., availability and affordability of healthcare, nutritious food, and physical activity), and the individual level (e.g., genetics, diet) to increase obesity risk^1^. Thus, solutions are needed at multiple levels to address high body weight and improve population health. Behavioral weight loss programs (BWL) are one of the gold-standard individual-level obesity treatments that teach individuals cognitive and behavioral skills for improving diet, physical activity, and managing weight. BWL produces weight losses of 5–10% and elicits improvements in diet quality, physical activity, cardiovascular disease risk factors, and reduced risk of developing diabetes and kidney disease^2,3^.

Deploying BWL in primary care is ideal given the large patient base and regular contact, and delivering these programs online reduces barriers to access^4,5^ Online BWL produces clinically meaningful weight losses in primary care when tested in efficacy and effectiveness trials^6–10^, but when they are implemented into routine care under real-world conditions, patients show lower rates of treatment uptake, completion, and outcomes^7^.

Successful online BWL implementation in primary care requires targeting multiple aspects of patient and healthcare provider behavior, including identifying eligible patients, presenting the program, enrolling patients, and fostering engagement. However, barriers complicate implementation at each stage and require tailored solutions. At the patient identification stage, primary care clinicians^11–13^ under-diagnose obesity^14,15^ and rarely address weight management during routine visits^16,17^. When providers do address weight, the risk of enacting weight stigma is high due to limited provider training in having patient-centered, stigma-sensitive conversations about weight^18^. Few patients follow through on referrals^19–21^, and when they do, non-usage attrition^22,23^ and substantial engagement dropoffs^21^ are common and limit health impacts^4,22–27^.

Controlled clinical trials have staff, time, training, and resources to identify, enroll, and manage patients in BWL; this is rarely true in primary care^28^. Likewise, outreach to patients in BWL improves weight loss outcomes, but this is uncommon in practice. Implementation strategies, including those related to training and monitoring, can improve the implementation of evidence-based interventions in clinical practice^29,30^. Successful implementation markers include clinicians’ adoption (uptake and use), acceptability, and feasibility within clinical practice^31^. To empirically investigate clinical practice integration implementation strategies, increase external generalizability, and maximize the likelihood of program success in real-world conditions, it is important to take a pragmatic approach.

A pragmatic research design includes both strengths and limitations. Strengths typically include high external validity, a large sample size, a simple research design, a focus on effectiveness over efficacy, and a focus on diverse healthcare settings. It often targets sustained implementation that relies on clinical staff and systems that can feasibly be left in place once the research concludes^32^. These strengths are balanced with certain tradeoffs. Oftentimes, clinical implementation partners are not research organizations, which can limit the type, quantity, and quality of data that are available for outcomes monitoring and evaluation. Fidelity monitoring may be limited due to the potential for reactivity, which can interfere with the goal of observing fully pragmatic implementation. This paper highlights the strengths and weaknesses of pragmatic designs, which play an important, unique role in the promotion of cardiovascular health and public health interventions to reduce cardiovascular risk.

The purpose of this study was to compare the random assignment of nurse care managers (NCMs) to two different clinical implementation strategy bundles on patient and NCM outcomes in an online BWL in a statewide primary care practice network. The Basic bundle (*Basic*) included instructions for enrollment and access to a list of enrolled patients. The Enhanced bundle (*Enhanced*) included the *Basic* features plus training to help NCMs engage and support patients and additional information on patient progress. The goals were to compare the effects of *Basic* versus *Enhanced* implementation on patient enrollment and completion at 3- and 12-month (co-primary outcomes), patient weight loss outcomes at 3-, 12-, and 24-month among all participants and treatment engagers, and clinician-rated program feasibility and acceptability (secondary outcomes). Based on available evidence that implementation strategies that target clinician training and patient monitoring are associated with evidence-based intervention uptake in clinical practice^33–35^, we hypothesized that NCMs randomized to *Enhanced* would enroll and retain more patients, their patients would achieve better weight loss outcomes, and they would report higher feasibility and acceptability compared to NCMs in *Basic*. Finally, this paper highlights the strengths and limitations of a pragmatic approach that relies on clinical partners for the implementation approach and outcomes assessment.

## Results

### Participants and setting

A total of 23 NCMs were recruited and trained (*n* = 12 *Enhanced*, *n* = 11 *Basic*), including 8 who were hired and trained mid-way through the trial to replace 14 who discontinued their employment; despite staffing changes related to the COVID-19 pandemic, all practices had NCM coverage. No NCMs withdrew from the trial while remaining employed by the community partner; changing employers made them ineligible for the study. The NCMs who provided demographic information (*n* = 19) covered 82 clinics, were all female, 84.2% White, and averaged 47.1 ± 15.7 years of age. On average, each NCM oversaw 2.9 clinics (range 1–5). Number of clinics overseen did not vary by implementation condition, *t*(21) = −0.89, *p* = 0.38.

Many aspects of treatment fidelity that were within the researchers’ control were regularly monitored. Proper randomization of NCMs was maintained throughout the study (e.g., when a new NCM joined the practice network and took over a retired NCM’s practices). All NCMs received the correct training, which was manualized to ensure continuity when new NCMs were trained. The training covered all planned material, there were no portal outages, and NCMs were able to log in throughout the study. Additionally, all emails to NCMs and letters for the Enhanced condition were sent as planned. However, fidelity in terms of NCM adherence to the study procedures after the training could not be directly assessed (e.g., how often Enhanced NCMs called participants to follow up on their enrollment experience).

### Patient outcomes

A total of 1765 patients were referred to the Rx Weight Loss program (RxWL). Of those, 41.8% (*n* = 738) completed the online consent, and 37.1% (*n* = 654) screened eligible and were enrolled in RxWL. More enrolled patients were referred by NCMs in *Enhanced* than *Basic* (490 [74.9%] vs. 164 [25.1%] patients). NCMs in *Enhanced* enrolled patients from more practices than *Basic* (26 vs. 17). NCMs in *Enhanced* enrolled a greater proportion of men (46.2% vs. 26.2%, *p* = 0.016) and a smaller proportion of individuals self-identifying as a member of a racial or ethnic minority population (4.9% versus 16.4%, *p* < 0.001) compared to NCMs enrolled in the *Basic* condition. There were no detectable differences between participants enrolled by *Enhanced* and *Basic* NCMs in mean age (*M* = 53.1, SD = 12.8 vs. *M* = 51.9, SD = 15.1, *p* = 0.358) or body mass index (BMI; *M* = 36.0, SD = 7.1 vs. *M* = 36.0, SD = 7.1, *p* = 0.983). There were no observed differences in the rate of completing the initial 12-week program in *Enhanced (*37.8% [*n* = 184]) versus *Basic* (37.6% [*n* = 62]) (*χ*^2^ = 0.003, *p* = 0.954). Similarly, 27.1% (*n* = 133) in *Enhanced* and 27.4% (*n* = 45) in *Basic* completed the maintenance phase at 12 months (*χ*^2^ = 0.005, *p* = .0.941). However, given the three-fold difference between the two implementation conditions in the number of patients enrolled, the number of patients who completed the treatment was greater in Enhanced vs. Basic (184 vs. 62 at 3 months and 133 vs. 45 at 12 months).

A subset of 540 participants had electronic health record (EHR) data available for analysis of weight change (405 in *Enhanced* and 135 in *Basic*; demographics in Table 1). There was no difference between *Enhanced* (82.0%) and *Basic* (82.3%) in the proportion of participants that had EHR data (*χ*^2^ = 0.004, *p* = 0.948). There was no statistically significant difference in mean ± SE weight loss for patients treated by NCMs in *Enhanced* and *Basic* after the 3-month program (2.95 ± 0.70 vs. 3.82 ± 0.44 kg, respectively; *t* = 1.057, *p* = 0.291). Likewise, there was no difference in the regain trajectory between *Enhanced* and *Basic* (*t* = 0.555, *p* = 0.579); regain was estimated to be 0.82 ± 0.27 kg in *Enhanced* vs. 0.65 ± 0.17 kg in *Basic* at the end of the 9-month maintenance intervention and 1.93 ± 0.63 vs. 1.52 ± 0.39 kg, respectively, at 24-month follow-up. In a subset analysis including only participants categorized as maintenance engagers (*n* = 253), weight regains trajectories again did not differ by NCM condition (*t* = 0.513, *p* = 0.608). Finally, although NCMs in *Enhanced* were encouraged to contact their patients more often, their patients had fewer EHR-documented clinical visits according to the number of weights in the EHR: patients managed by NCMs in *Basic* had more weights in the EHR over the 2-year study period (*M* = 5.44, SD = 2.65, *n* = 128) than patients managed by NCMs in *Enhanced* (*M* = 4.65, SD = 2.52, *n* = 412; *p* = 0.002).

**Table 1:** Baseline characteristics of participants with EHR data available (*N* = 540) and without EHR data available (*n* = 114)

Characteristic	Analysis sample	Excluded from Analysis
	*N* = 540	*n* = 114
*Gender*—*N* (%)		
Female	384 (71.1%)	76 (66.7%)
Male	156 (28.9%)	38 (33.3%)
*Race*—*N* (%)		
American Indian/Alaska Native	1 (0.2%)	2 (1.8%)
Asian or Pacific Islander	8 (1.5%)	1 (0.9%)
Black or African American	13 (2.4%)	6 (5.3%)
White	509 (94.3%)	102 (89.4%)
Other	9 (1.7%)	3 (2.6%)
*Ethnicity*—*N* (%)		
Hispanic/Latinx	15 (2.8%)	2 (1.8%)
Non-Hispanic	525 (97.2%)	112 (98.2%)
Age—*M* (SD)	52.8 (13.4)	52.1 (12.8)
Body mass index (kg/m^2^)—*M* (SD)	36.0 (7.1)	36.8 (6.0)

*M* mean, SD standard deviation.

### NCM outcomes

Although participation was voluntary, 100% of NCMs consented to take part in the study, signifying complete adoption. NCMs who completed the feasibility and acceptability survey (*n* = 19) rated RxWL as being acceptable and feasible (2.0 ± 0.52 and 1.6 ± 0.50 on a scale of 1 [strongly agree the intervention was acceptable or feasible] to 5 [strongly disagree], respectively, indicating average Likert scale ratings of agree to strongly agree). NCMs in *Enhanced* (*n* = 11) had a mean acceptability rating of 2.0 ± 0.59 and a mean feasibility rating of 1.6 ± 0.51, whereas those in *Basic* (*n* = 8) had a mean rating of 1.9 ± 0.44 for acceptability and 1.7 ± 0.50 for feasibility, indicating similarly high ratings.

## Discussion

This study examined whether providing primary care clinicians with different support for implementing an online BWL within routine care influenced enrollment, completion, weight change, and clinician acceptability and feasibility. Few studies have directly compared implementation strategy intensities on patient and healthcare provider outcomes; fewer have done so within a pragmatic trial. NCMs who received *Enhanced* strategies enrolled nearly three times more patients than NCMs who received *Basic*, but there were no differences in treatment completion, weight change, or NCM satisfaction.

The clinical implementation partner was not able to report how many patients were eligible for or referred to RxWL by *Basic* versus *Enhanced* NCMs. Nevertheless, nearly 75% of enrolled patients were enrolled by NCMs in *Enhanced* (490 vs. 164 patients), suggesting that *Enhanced* strategies may have improved NCMs’ ability to enroll patients. Additionally, NCMs who received the *Enhanced* training and dashboard enrolled a greater proportion of men but a smaller proportion of individuals who self-identify as members of a racial or ethnic minority background; future research should continue to investigate how implementation strategies can best support referral to, enrollment in, and support during BWL for individuals from racial and ethnic minority backgrounds. It is critical to use implementation strategies to reduce health disparities by supporting equitable opportunities for enrollment in ineffective programs. The research team specified a goal of maximizing recruitment of men and people from racial and ethnic minority groups in the trial since these patient groups are under-connected with and under-served by BWL programs though enrollment through primary care is a challenge across all patient groups^36,37^. Engaging primary care patients in obesity treatment is challenging: one recent study showed that only 9.9% of eligible patients enroll^19^. This trial’s enrollment rate was 37%: *Enhanced* led to more treatment completion (133 from *Enhanced* vs. 45 in *Basic* at 12 months) and, therefore, a greater total number of people impacted and pounds lost. Implementation support thus may have influenced treatment reach but not completion. This was surprising because non-usage attrition^22,23^ and diminishing engagement^21^ are common^4,22–27^, so NCMs in *Enhanced* received recommendations to call patients after 2 weeks, weekly emails identifying declining engagement, and monthly progress reports to help at-risk patients re-engage. Yet completion rates were nearly identical across conditions: 38% of enrolled patients completed the 3-month phase, and 27% completed the 9-month phase.

Weight change outcomes were similar. Irrespective of NCM assignment, patients displayed comparable weight changes at 3-, 12-, and 24-month, suggesting no benefit of *Enhanced* implementation on completion or weight loss. NCMs in *Enhanced* may not have had time for recommended follow-up or patient data review^28^. The RxWL portal’s separation from NCMs’ EHR workflow may have exacerbated this barrier, and this should be evaluated in future primary care pragmatic trials^38^. Implementation strategies for this setting require further investigation.

NCM-level outcomes were encouraging: both groups gave strong, similar acceptability and feasibility ratings, despite NCMs in *Enhanced* being asked to contact patients and review data more frequently. *Enhanced* support’s apparent effects on patient enrollment (in terms of the number of participants who enrolled and completed) alongside strong satisfaction ratings are promising. All NCMs adopted the program by consenting to participate^39^.

Study strengths include the pragmatic approach in a statewide primary care network, which allows for measuring real-world implementation factors, uptake, and engagement. Pragmatic designs help researchers envision a realistic implementation of their programs, empirically evaluate implementation strategies, and identify barriers to sustainability. Other strengths include a grounding in implementation theories^30,40^ and a direct comparison of two implementation strategy bundles, providing insight into the differential utility of implementation approaches on obesity treatment uptake in primary care. Use of an evidence-based, fully automated BWL that promotes cardiovascular health through lifestyle change is also a strength, given the low cost and high scalability potential.

The study also has limitations, some of which are related to the pragmatic design. For example, regular fidelity checks of NCM adherence to the protocol were explicitly not included in this trial because the goal was to study real-world, pragmatic implementation. Presumably, this would not likely include fidelity checks. Recent work has described a Framework for Implementation Fidelity, which may be useful for evaluating fidelity, when possible, in future pragmatic trials^41^. In addition, the practice network could not provide a separate count of patients referred by implementation condition, and these participants did not provide consent to research and were, therefore, unknown to the researchers, which precluded statistical comparisons of enrollment rates by condition or practice location. Lack of access to these data is a tradeoff inherent to the pragmatic nature of the study. This is a factor for consideration for other research teams working with community partners. Secondly, the community partner experienced unexpected constraints that prevented them from providing long-term follow-up health outcomes, which would have strengthened the effectiveness analysis and would have added contextual detail to the impact of the program implementation in this setting. Long-term follow-up of implementation is highly recommended, given changing factors in the implementation setting that may lead to de-implementation or reduced treatment fidelity. The referral process and ongoing participant management were not integrated into the NCMs’ routine patient management platforms, which changed during the study and varied across sites; it is unknown how these aspects, which commonly occur in pragmatic research, might have affected implementation. Additionally, the use of the EHR naturally resulted in missing data that impeded inclusion in clinical outcomes analyses (n = 114)^42^. It is important to note that weight outcomes in behavioral studies often follow a dose–response relationship: training time was constant across all groups, but the research team could not evaluate how much time NCMs spent on the referral process and on subsequent contact with their patients. Further, at the clinicians’ suggestion, their portal consisted of a dashboard showing participant information immediately upon loading. This facilitated usability but inhibited the ability to track clinician portal use since it did not require monitorable interaction (i.e., clicks). Finally, BWL’s sole focus on individual-level behavior change is inherently limiting for addressing obesity at the population level because it does not address societal or community factors, important areas for obesity treatment research.

A few limitations and suggestions for future research are linked to the trial’s effectiveness aim. Patient outcomes analyses are limited by sample homogeneity (mostly non-Hispanic white women). Future research should include long-term follow-up of health outcomes as well as consideration of how weight stigma and discrimination affect these outcomes. Additionally, given the elusiveness of long-term weight maintenance in fully online programs, the sustainability of both program implementation and weight maintenance remains an area for future research.

This pragmatic trial’s findings showed that an *Enhanced* implementation strategy bundle helped more patients enroll in the BWL. NCMs found the program to be acceptable and feasible. The findings highlight the advantages and limitations of pragmatic implementation research involving obesity treatment in primary care. Future research must identify strategies to enhance enrollment in evidence-based weight loss programs, program completion, and weight outcomes in primary care with the goal of reducing cardiovascular risk, upholding patient autonomy, and improving quality of life and overall health through multiple behavior changes. Researchers must continue to collaborate with community and clinical partners to maximize successful implementation, improve systems to allow for complete data capture, and ultimately reduce cardiovascular risk in real-world clinical settings.

## Methods

### Study design and setting

This manuscript focuses on implementation outcomes from a pragmatic hybrid type 2 implementation-effectiveness trial that enrolled participants between May 2018 and March 2021 (until the target sample size was achieved); data collection concluded in 2022. NCMs completed feasibility and acceptability measures in August 2020, ~2 years after most clinicians were trained. The protocol, 3-month weight loss outcomes, and 12- and 24-month weight loss outcomes by maintenance condition (newsletter controls or two active treatment schedules [lessons delivered monthly or in bursts]) are presented elsewhere^43–45^. The trial was conducted in partnership with [Rhode Island Primary Care Physicians Corporation], a primary care practice organization of >60 primary care practices across [Rhode Island, USA, that utilizes a Patient-Centered Medical Home model^46,47^. The study was conducted in accordance with the Declaration of Helsinki and approved by the lead author’s Institutional Review Board. All patients provided written electronic informed consent. The study was prospectively registered on clinicaltrials.gov (NCT03488212). This report follows the Consolidated Standards of Reporting Trials (CONSORT).

### Participants

**Fig. 1:**
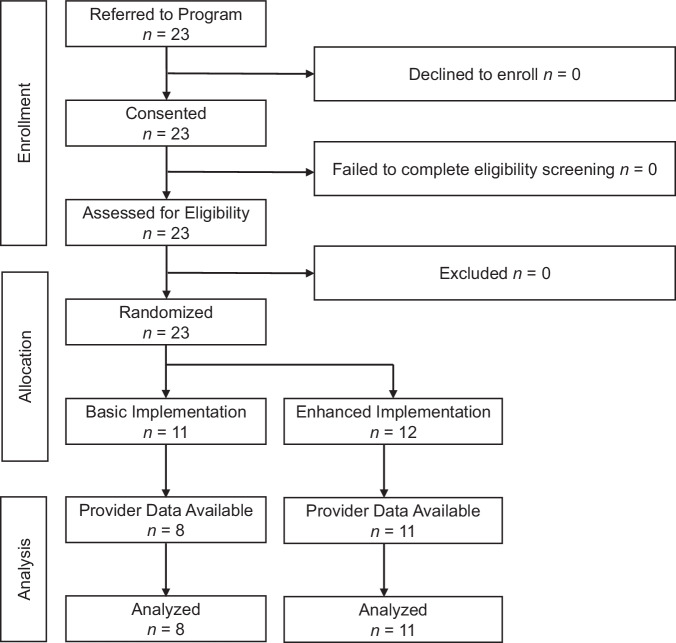
Flow of nurse care managers through the trial*.* Clinicians in Rhode Island were recruited for and participated in a hybrid implementation-effectiveness pragmatic trial of two implementation strategy bundles to facilitate patient participation in an online behavioral weight loss program between 2018 and 2022.

**Fig. 2:**
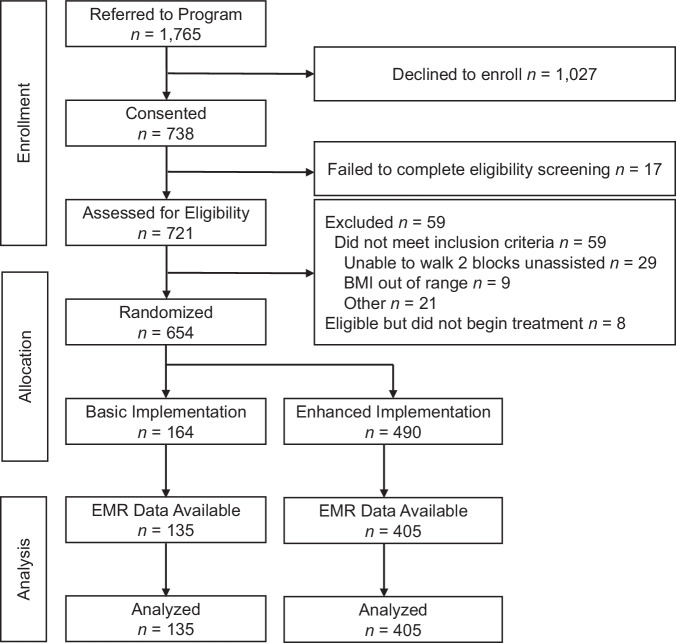
Flow of participants through the trial*.* Primary care patients in Rhode Island were recruited and participated in a hybrid implementation-effectiveness pragmatic trial of an online behavioral weight loss treatment between 2018 and 2022.

NCMs (Fig. 1) and their patients (Fig. 2) participated. All NCMs employed by [Rhode Island Primary Care Physicians Corporation] were eligible. RxWL was presented to NCMs as an organization-wide initiative, but participation was voluntary. All NCMs completed informed consent procedures and were then randomized to *Basic* or *Enhanced* training.

[Rhode Island Primary Care Physicians Corporation] Patients between ages 18 and 75 with a BMI > 25 kg/m^2^ who were deemed medically appropriate by the lead of their primary care medical team (e.g., a primary care physician), had an Internet-connected device (i.e., smartphone, computer, tablet) access, and were able to exercise safely by walking 2 blocks without stopping were eligible for the study. These inclusion criteria were modified from the original protocol to fit the community partner’s needs: an upper BMI limit was removed, the age maximum was increased from 70, and the exercise safety requirement was modified to allow the use of an assisted device.

Exclusion criteria included: taking weight loss medication; on a liquid weight loss diet; pregnant, lactating, <6 months postpartum, or plan to become pregnant within 12 months. Per this trial’s pragmatic nature, NCMs managed recruitment; patients had no interaction with research staff unless they required technical assistance that NCMs could not provide.

### Interventions

#### Weight loss

RxWL is an evidence-based online BWL that combines online skills training lessons, self-monitoring of calorie intake, physical activity, and body weight, and tailored feedback^48^. All patients received the same initial 3-month program. Participants were randomized to one of three 9-month maintenance conditions that involved 9 online skills training lessons with brief self-monitoring periods on two different schedules or a newsletter control; all participants received the same number of maintenance lessons/newsletters, and the lessons were the same length of time^43^. EHR weight change data were collected through the 12-month program and after an additional 12 months of no-intervention follow-up (i.e., 24 months). Maintenance conditions details are reported elsewhere^43^.

#### Implementation

Two implementation strategy bundles focused on NCM training and digital tools were tested. Both bundles included the following evidence-based implementation strategies: identifying champions, centralizing technology assistance, identifying barriers and facilitators, ongoing educational outreach visits and training, developing and distributing educational materials, facilitating clinical data relay to clinicians, organizing clinician implementation team meetings, and reminding clinicians^30^. Implementation strategy bundles were designed to impact NCM feasibility and acceptability by targeting implementation determinants at the innovation and inner setting level of the Consolidated Framework for Implementation Research^40^. Specifically, the training and electronic tools were designed to present RxWL as credible, evidence-based, and adaptable to patients’ needs as well as to enhance the information technology infrastructure, promote the compatibility of RxWL within existing workflows, and increase access to knowledge and information supportive of RxWL delivery within [Rhode Island Primary Care Physicians Corporation].

##### Basic training and tools

NCMs in *Basic* received a one-time 60-min live training that included an explanation of RxWL’s scientific underpinnings, instruction for participant enrollment, and troubleshooting. *Basic* also provided access to a clinician portal listing patients enrolled in RxWL with start date, date of birth, practice affiliation, program week, and randomization to weight loss maintenance condition. The portal contained troubleshooting help for common issues. The portal was hosted on a website separate from the electronic health record system that the community partner used, thus requiring a separate login; there was no integration between their electronic health record and the clinician or patient study portals. The training included a discussion of provider bias in referral to and support in BWL programs, respecting individual autonomy to refuse the program, how joining a weight loss program may or may not support overall well-being for an individual at that time, and acknowledging structural barriers to health management. NCMs in *Basic* received a weekly email reporting study-wide progress towards the enrollment goal and a link to their portal.

##### Enhanced training and tools

*Enhanced* added to *Basic* by training NCMs to provide patients with a study overview and to tailor their program recommendation to patients’ unique barriers, values, and goals. To facilitate effective recruitment and retention, NCMs were trained in motivational interviewing skills for addressing enrollment and engagement barriers and ambivalence about joining a structured program, as well as training to support engagement via a focus on patents’ goals and values^30^. It included a brief but more detailed review of structural determinants of health as they pertain to weight status. The entire training was 60–90 min long. This supplements NCM's existing efforts to support healthy weight management in patients seeking to reduce body weight, which was a stated organization-wide priority. NCMs were asked to call patients 2 weeks after referral for a brief check-in to evaluate clinical or technical support needs and provide encouragement.

Compared to *Basic, the Enhanced* clinician portal included additional flags for non-adherence (missed lessons or self-monitoring; slow or too rapid weight loss), suboptimal goal achievement, date of last login, total and weekly weight loss, and practice summaries showing patients enrolled and adherence for each practice. The weekly email provided to NCMs in *Enhanced* additionally included enrollment progress for the NCM’s practices compared to all other practices and a list of patients for follow-up due to non-adherence. These patient monitoring strategies support the integration of RxWL into routine clinical practice by allowing for patient progress monitoring rather than simply acting as a referral resource.

For NCM in *Enhanced*, a progress letter detailing engagement and weight losses was sent online and by mail to the NCM, patient, and their primary care physician at weeks 4, 8, and 12.

### Protocol

One Principal Investigator (J.G.T.) generated and managed the random allocation sequence used for NCM randomization, which matched NCMs on practice setting (rural versus urban) and size. The pairs were then randomized in a 2 × 2 design with a 1:1 ratio to receive *Basic* or *Enhanced*. Each NCM received one randomization for all their practices, acting as a nested arrangement within each NCM. Since randomization was assigned to NCMs, those who switched clinics retained their randomization assignment. When NCMs left and new NCMs took over their practices, their replacement was assigned to their predecessor’s condition. NCMs and investigators were not masked to condition assignment. Participants were initially blinded to maintenance condition assignment.

Two clinical psychologists (one Co-Principal Investigator and one Co-Investigator) enrolled and trained NCMs separately by implementation condition. NCMs received a list of their practices’ eligible patients based on age and BMI who had scheduled appointments in the next week. NCMs introduced the program to appropriate patients, confirmed eligibility, and documented referrals in the EHR. They provided a unique code that gave patients access to an informational video, enrollment screening, informed consent process, and the treatment portal. The research team conducted regular calls during an NCM standing meeting with a single randomization group (*Enhanced* or *Basic*) to evaluate implementation and address questions. NCMs were asked to refrain from sharing information that could differ between randomized groups. Investigators involved in outcome assessment were masked.

### Outcome measures

#### Patient contact and enrollment

The practice network provided the total count of referrals to RxWL. We calculated the proportion that enrolled following referral.

#### RxWL program completion

The percentages of enrolled patients who completed the initial 3-month and maintenance programs were calculated. Initial program completion was defined as accessing the week 12 lesson and/or recording any self-monitoring data during week 12. Maintenance completion was defined as accessing at least 4 lessons and/or recording any self-monitoring data for at least 30 days^44^.

#### Patient weight loss by NCM implementation condition

**Fig. 3:**
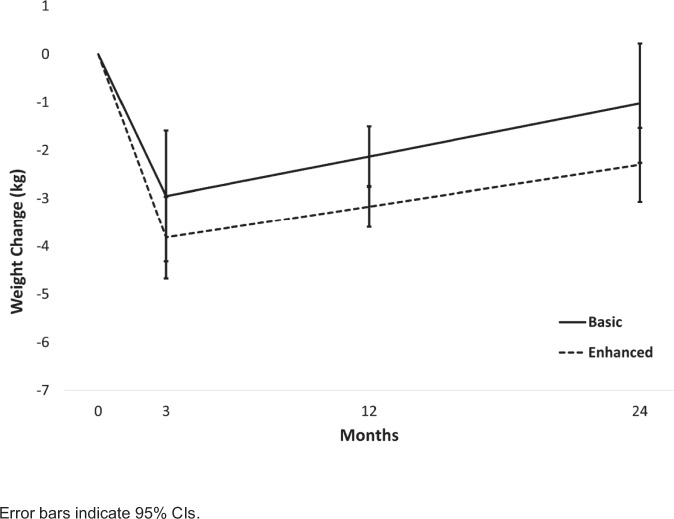
Intent-to-treat participant weight change by nurse care manager assignment. Modeled participant weight change (kg) over the 2-year study period for the intent-to-treat analysis (*N* = 540) by Nurse Care Manager assignment.

We compared weight loss outcomes at 3 months and weight regain at 12 and 24 months for all participants available in the EHR (intent-to-treat approach; ITT; Fig. 3) and for program engagers (defined as viewing at least one lesson or entering self-monitoring information at least once during the 9-month maintenance period), as well as the number of weights available in the EHR, by their NCM’s condition assignment.

#### Feasibility and acceptability

Percent of NCMs that enrolled signifies adoption, an implementation measure. NCMs also completed a questionnaire using a 5-point Likert scale (1 = strongly agree, 2 = agree, 3 = neutral, 4 = disagree, 5 = strongly disagree) to assess the feasibility (5 items) and acceptability (6 items) of the RxWL program. Feasibility and acceptability of an innovation are two key implementation outcomes that are an indicator of implementation success^31^. One component of satisfaction is acceptability, which focuses on the dynamic experience with a specific intervention, but these terms are commonly used interchangeably^31^. Feasibility items addressed usefulness, ease of use, and fit of the program in the existing workflow and setting and acceptability items addressed observed effectiveness and relative advantage of RxWL over the existing standard of care. Items were averaged and lower scores signified greater feasibility and acceptability.

### Statistical plan

Analyses were conducted using IBM SPSS Statistics 26. Descriptive statistics were computed for participant characteristics. Independent samples *t*-tests were used to compare NCM characteristics (e.g., demographics, caseloads) by implementation condition. Analysis examining 2-year weight change was conducted via piecewise linear mixed effects models using maximum-likelihood estimation. The analysis followed the ITT principle and included all participants with any weight information available in the electronic medical record. The model included a knot at 3-month (i.e., end of the 3-month weight loss phase and beginning of the maintenance phase) and a fixed linear effect of time. A dummy coded effect indicator for implementation condition was allowed to interact with the time effect to test for differential trajectories in weight from 3 months to 2 years. Covariates of patient age, sex (male/female), and identification with a racial/ethnic minority group (yes/no) were included as fixed effects. Random intercepts accounted for the nesting of observations within individuals. Least-squares mean estimates of weight loss were calculated.

The parent study’s sample size (*n* = 600) was determined based on power to detect weight loss outcomes based on maintenance conditions (the co-primary aim—data presented elsewhere) with similar effects estimated for implementation outcomes^44^.

## Data Availability

Deidentified study data representing reported findings may be available on reasonable request from the Co-Principal Investigator, JGT (jthomas4@lifespan.org).
